# Observational study exploring the association between sexes and perioperative morbidities after aseptic hip revision arthroplasty

**DOI:** 10.1186/s12891-026-09607-1

**Published:** 2026-02-21

**Authors:** Greta Drews, Filippo Migliorini, Christian Götze, Julian Koettnitz

**Affiliations:** 1 Ruhr University Bochum, University Hospital Auguste-Viktoria Department of General Orthopaedics, Am Kokturkanal, 32545 Bad Oeynhausen, Germany; 2 Department of Orthopaedics and Trauma Surgery, University Clinic Aachen, RWTH Aachen University Clinic, 52064 Aachen, Germany

**Keywords:** Blood transfusion, Complications, Sex differences, Revision THA

## Abstract

**Background:**

Sex-based analyses in total hip arthroplasty have become increasingly important in understanding the nuanced outcomes and risks associated with this surgical procedure. Recent studies analyzed the complex interplay between sex-specific factors and revision rates, challenging previously held assumptions about sex disparities in hip replacement outcomes. This study examines the correlation between sex and the occurrence of perioperative morbidities after aseptic hip prosthesis revisions.

**Methods:**

Data from 261 patients following aseptic revision THA (total hip arthroplasty) were collected. The occurrence of systemic and surgery-related complications during the hospitalisation and in the follow up, the units of blood transfused, and the change in Hb (hemoglobin) were investigated. Hb was collected preoperatively and at 1, 2, and 4 days postoperatively. Analyses of sex differences between different BMI and age groups for perioperative complications and transfusion rates was carried out. The Fischer Exact test and the independent t-test were mainly used for the investigations.

**Results:**

There were significant sex differences in the length of stay (12.10 ± 4.60 vs. 13.90 ± 5.50; *p* = 0.007, Cohen’s d = 0.344), the BMI in elderly patients (28.30 ± 4.9 vs. 25.80 ± 4.4; *p* = 0.003; d = 0.553) and occurrence of multiple surgical complications (0.12 ± 0.35 vs. 0.27 ± 0.76; *p* = 0.024, d = 0.243), esp. delayed wound healing (*n* = 0 vs. *n* = 7; *p* = 0.046, phi = 0.131). Furthermore, occurrence of anemia (*n* = 9 vs. *n* = 54; *p* = < .001, η2 part = 0.092), and transfusion rate in elderly patients were significantly higher (0.20 ± 0.62 vs. 1.06 ± 1.83; *p* = 0.001, d = 0.642). Significant sex differences were found for surgical complications in the subgroup of BMI (Body-mass-Index) under 30 kg/m^2^ (0.12 ± 0.373 vs. 0.31 ± .84; Cohen’s d = 0.277; *p* = 0.027). No sex differences were found for multiple systemic complications (0.11 ± 0.44 vs. 0.13 ± 0.42, *p* = 0.635).

**Conclusions:**

Although this study found differences in the sex subgroups in terms of age and BMI, no general sex differences between men and women could be identified. When considering perioperative and follow-up risks, sexes do not play a crucial role.

**Trial registration:**

Not applicable

## Background

Sex-based differences in medicine have become a widespread research focus. Significantly influences the consequences of health and diseases in the Western world. A recent example for sex differences is represented by the COVID pandemic [1]. In orthopedic surgery the demand for primary and revision THA procedures is expected to rise with an aging and more active society [2]. Sex-based differences in orthopedic surgery complications may reflect anatomical, physiological, behavioral and social factors. Clinically, women tend to have higher perioperative complication rates, including delayed wound healing, increased surgery-related complications [3, 4]. For example, postmenopausal osteoporosis predisposes them to higher intraoperative fractures [5]. Conversely, men tend to experience earlier revision surgery, which may reflect differences in rehabilitation, activity levels, or biological responses to implants [6]. However, Chen et al. 2021 did not find any significant sex differences in revision rates after primary total hip arthroplasty after 2-years of observation. By analyzing a subgroup of young patients under 55 in their study, they could show an increased risk of revision surgery for younger women [7]. Recently, women were reported to have a greater preoperative impairment in mobility, higher pain levels before surgery and increased anxiety which could influence the surgery and possible complications [8].

While sex differences provide critical insights into patient-specific risks, these must be understood in the context of other influential factors, such as body mass index (BMI) and age. These factors have additive and individual effects on orthopedic surgery outcomes and can influence subsequent complications, thereby modulating the effect of sex alone.

For example, several studies have demonstrated a correlation between BMI and increased complication rates, such as infection rate and prolonged rehabilitation [9–11]. However, more recent findings indicate that withholding surgery in patients with high BMI offers only minimal benefit. For instance, DeMik et al. 2022 found out, that the complication rate increases marginally, compared to the successful surgery in the groups with BMI of > 40 kg/m^2^ and 50 kg/m^2^ [12, 13]. The influence of age in complications after hip and knee arthroplasty was shown in a large epidemiological registry study analyzing over 2.8 million arthrosplasty patients. Furthermore, it was shown, that older female patients tended to have higher comorbidity burdens affecting postoperative recovery[14, 15].

This interconnected influence of sex, BMI, and age in arthroplasty emphasizes the need for integrated patient assessment and tailored perioperative management. Understanding these factors will aid clinicians in optimizing surgical planning, minimizing complications, and improving individualized rehabilitation strategies, as an increase of 43–71% in total hip and knee arthroplasty is expected, which will proportionally increase the number of patients with perioperative complications [16–22].

To our knowledge, a sex study of aseptic revision THA has not yet been conducted. The primary investigation of this analysis was, whether a difference in the perioperative and follow-up surgery-related and perioperative systemic complications between men and women exist. Additionally, an exploration of factors associated with aseptic revision THA between males and females was done. The second goal was to analyze sex differences in connection to high age or BMI. The first hypothesis was that sex differences in perioperative and follow-up complications appear and the second hypothesis was that sex differences for perioperative and follow-up complications in high age or BMI subgroups would occur.

## Methods

### Study design

The present study was performed according to Strengthening the Reporting of Observational Studies in Epidemiology (STROBE) [23]. This study was conducted at the Department of Orthopedic Surgery of a University Hospital in Germany. The study was conducted in accordance with the Declaration of Helsinki and approved by the local Ethics Committee of the university hospital.

### Study protocol

Data from patients who underwent aseptic revision THA during 2018 to 2021 were retrieved. The data were retrieved using Pegasos 7 (Nexus Marabu GmbH, Berlin, Germany) and collected in Microsoft Excel (Microsoft Corporation, Redmond, WA, USA). The following data were collected at admission: age, sex, side, body mass index (BMI), length of hospital stay, length of intensive care unit stay, and American Society of Anesthesiologists physical status (ASA) [24]. During hospitalization, the following data were collected: complete hip replacement or partial component replacement (femoral or acetabular replacement), preoperative and postoperative hemoglobin (Hb), the incidence of systemic and surgical complications, and the frequency of blood unit transfusions. Systemic complications included pulmonary, cardiac, urogenital, and neurologic complications. Surgical-related complications included early infections, neurologic disorders, fractures, bleeding, aseptic loosening, surgical interventions post-surgery, and post-discharge complications such as infections or instabilities. If patient data was not accessible, the patient was excluded from the present investigation.

### Eligibility criteria

All patients undergoing aseptic revision THA were retrieved, and their eligibility was assessed. The inclusion criteria were: (1) Patients with primary uncemented THA; (2) patients aged between 40 and 100 years; (3) accessible patient data; and (4) revision surgery. The exclusion criteria were: (1) septic revision surgery; (2) any blood abnormalities (sickle cell anemia, thalassemia, leukemia, lymphoma); and (3) a peripheral arteriovenous or neurologic ailment (Peripheral arteriovenous malformation (AVM), Parkinson’s disease, epilepsy).

### Perioperative management

Revision arthroplasties were conducted with Zimmer Biomet Zweymüller stem and TM system (Zimmer Biomet, Warsaw, IN, USA) or REDAPT Acetabular System with POLARCUP dual Mobility system (S&N GmbH, London, UK) in combination with the shaft system MUTARS RS (Implantcast GmbH, Buxtehufe, DE). All revision components were implanted cementless, except the POLARCUP in special occasions. For the cups the individual decision for screws (at least two screws) was made in depending on the situation during surgery. When implanting large cup sizes from the REDAPT Acetabular System, the Polarcup was cemented into the cup for better dislocation security. No tranexamic acid was given, because it was not a standard practice in the hospital during the time of data collection. For the THA an anterolateral, dorsal, and lateral approach was used. After surgery, patients with aseptic revision THA received diclofenac 75 mg for 10 days to prevent heterotrophic ossification. A stationary or ambulant rehabilitation program was organized prior to hospital release. Patients in an ambulant rehabilitation program spend the day at the clinic and then go home at night. Patients in a stationary rehabilitation program spend both the day and night at the clinic.

### Blood unit supply

The indication for the blood unit transfusion was according to the restrictive Cochrane guidelines: Hb-levels over 8.0 g/dL indicated no transfusion; between 7 and 9 with concomitant clinical symptoms such as dizziness, nausea, malaise, or loss of appetite; and Hb-levels under 8 g/dL indicated transfusion [25].

### Statistical analyses

All statistical analyses were performed using the software IBM SPSS version 28 (IBM, Armonk, NY, USA). An a-priori power analyses was done using all collected variables and more than three predictors, which set the level of patients to be examined to *n* = 135 [26]. Metric-scaled data were analyzed by mean, standard deviation, and variance. Nominal, dichotomous data were analyzed by Fisher’s exact test. Age, sex, BMI, length of hospitalization, pre- and postoperative Hb, and frequency of transfusion were listed metrically. Sex, systemic, and surgical complications were listed nominally. ASA score and preconditions were listed ordinally. Prior to statistical analysis, the distribution of the continuous outcome variables was assessed for normality. Data meeting the criteria for normal distribution were analyzed using parametric tests, like the independent T-Test while non-normally distributed data were analyzed using appropriate non-parametric methods, like Mann–Whitney-U-Test or Spearman’s Test. This ensured that statistical assumptions for subsequent analyses were met. For the analysis of metric and nominally scaled variables, the T-test for independent samples, variance analyses, the Levene test, and the Welch tests were used. Cohen’s d (small 0.20; medium 0.50; large 0.80) and 95% interval were used as effect sizes. The effect size used was phi (small 0.10; medium 0.30; large 0.50). The significance level was set to two-sided with α = 0.05.

## Results

### Recruitment process

In total, data from 281 patients with aseptic revision THA were retrieved from 2018 to 2021. In 20 patient cases the data was not fully accessible due to a lack of documentation.

#### Follow-up recruitment process

187 patients appeared to the unscheduled follow up. Of these, 54 were men and 133 were women. The mean follow-up-time were 248 days ± 321 days. The minimum follow-up time was 6 days, the maximum 1496 days (d). The follow up time between men (mean 114.22 (d)) and women (mean 302.76 (d)) was significantly different (*p* = 0.001, 95%CI −259.04—118.02).

### Patient demographics

In General, no significant difference was found between preoperative and postoperative range of motion (*p* = 0.358, Z = 0.918/*p* = 0.001, Z = 9.413). A significant difference was found between pre- and postoperative visual pain scale (*p* = 0.001, Z = −0.9413). For the mobility (1 = unlimited, 2 = 500 m, 3 = 50 m, 4 = in-room mobile, 5 = immobile) a significant difference was shown between the pre- and postoperative situation. While the number of immobilized patients decreased by 92% (50 to 4 patients), the unlimited postoperative mobility was reduced by 50% (12 to 6 patients). In addition, postoperative 50 m (room level) mobility increased significantly (111 to 165 patients, *p* = 0.001, correlation coefficient = 0.279). Overall, male patients were more mobile postoperatively than female patients (middle rank 119.32 to 136.20, Z = −1.999, *p* = 0.046). In relation to walking assistants (none, crutches, walker, wheelchair, immobility) a significant difference from pre- to postoperative was shown. Especially the use of wheelchairs and the immobilization was clearly reduced (pre to post: 6.5% to 3.5%/14.6% to 0.8%, *p* = 0.001, phi = 0.563).

No highly significant sex differences were found, either for the indication or for parameters such as pain or ROM. The subgroup age analysis revealed only minor differences in BMI and length of stay. To see statistical values of demographic parameters, please see Table 1.

**Table 1:** Patient demographics

Demographics	Men	Women	P, statistical Effect
THA group (sex)	38.3% (100 of 261)	61.6% (161 of 261)	
ASA score	2.1 ± 0.7	2.0 ± 0.6	0.503, Z = −0.670
cCRA	37.0% (37 of 100)	36.0% (58 of 161)	0.144, phi = −0.91
pCRA	63.0% (63 of 100)	64.0% (103 of 161)	0.190, phi = 0.086
- Stem + Head/Inlay	18.0% (18 of 100)	4.34% (7 of 161)	0.002*, phi = 0.260
- Cup + Head/Inlay	33.0% (33 of 100)	34.7% (56 of 161)	
- Head/Inlay	11.0% (11 of 100)	20.4% (33 of 161)	
- Head	1.0% (1 of 100)	4.3% (7 of 161)	
Indication for replacement:			0.337, phi = 0.163
Chronic Pain	*n* = 50	*n* = 61	
Instability of the components	*n* = 20	*n* = 40	
Chronic pain & Instability of components	*n* = 18	*n* = 27	
Periprosthetic fracture	*n* = 12	*n* = 30	
Postoperative Motor dysfunction of foot Flexion and Extension	*n* = 0	*n* = 3	
Age	71.8 ± 12.2 years	70.16 ± 12.9 years	0.304, d = 0.130
BMI	28.54 ± 4.6 kg/m^2^	26.84 ± 5.09 kg/m^2^	0.611, d = 0.065
Pre-diseases	2.4 ± 1.9	2.1 ± 1.6	0.299, d = 0.132
Surgery time	106 ± 47.3 min	100 ± 39 min	0.259, d = 0.323
The length of hospital stay (d)	12.1 ± 4.6	13.9 ± 5.5	0.007*, d = 0.344
ROM Preoperative	89.1 ± 14.1	89.5 ± 17.7	0.910, d = −0.022
ROM Postoperative	88.1 ± 7.6	87.5 ± 7.5	0.737, d = 0.044
VAS Preoperative (pain)	6.7 ± 0.1	6.9 ± 0.1	0.265, d = −0.147
VAS Postoperative (pain)	1.8 ± 0.7	2.0 ± 0.8	0.149, d = −0.267
≥ 75 years BMI	28.3 ± 4.9	25.8 ± 4.4*	0.002*, d = 0.558
≥ 75 years length of hospital stay (d)	12.9 ± 5.3	15.1 ± 4.6*	0.016*, d = 0.434
≥ 75 years surgery time	115 ± 50.2 min	104 ± 41.4	0.196, d = 0.231
≥ 75 years Pre-diseases	2.7 ± 1.8	2.6 ± 1.8	0.852, d = 0.033

*THA* Total hip arthroplasty, *cCRA* complete component revision arthroplasty – full change of stem, cup, Head/Inlay, *pCRA* partial component revision arthroplasty

^*^significantly different; (d) = days; 100 = male patients; 161 = female patients; 261 = all patients; ROM = range of motion; *VAS * visual analogue scale for pain, Z = Mann-Whitny-U-Test effect strength, d = Cohen’s d effect strength, phi = chi square effect strength

The paprosky score was documented in 27 male and 29 female patients. In male patients type 2a to 3a were detected (9, 9, 2, 7 Patients) and in female patients type 2a to 3b (9, 5, 6, 3, 9 patients). No significant differences could be found between the paprosky types in the postoperative outcome for the range of motion, the visual pain scale or surgical complications or the type of revision surgery in the follow-up.

### Complications

#### Perioperative

There were 12 detected surgery-related complications in 11.0% (11 of 100) male patients and 41 counted surgical complications in 16.1% (26 of 161) female patients (Table 2). There were 22 systemic complications in 3.1% (5 of 161) male patients and 11 systemic complications in 3.0% (3 of 100) female patients (Table 3).

**Table 2:** Perioperative surgery-related complications of men and women after aseptic revision surgery; total number of surgery-associated complications for men was *n* = 12 and for women *n* = 41

Surgery-Related Complications	Men (n)	Women (n)	p, statistical effect (phi)
Intraoperative injuries			
- Vascular injuries	2	11	0.525, 0.069
- Nerve injuries	0	2	0.140, 0.108
- Fractures	5	5	0.425, 0.150
Surgery-associated infections			
- Periprosthetic infections	0	1	1.0, 0.049
- Wound healing disorder (> 7 d)	0	7	1.0, 0.008
Postoperative fractures			
- Periprosthetic fracture	0	3	0.288, 0.085
Postoperative loosening			
- Aseptic loosening	1	0	0.383, −0.079
Postoperative neurologic disorders			
- Paranesthesia/weakness of movement	4	12	0.357, 0.121

Nerve damage that occurs during surgery must be considered separately from postoperative neurological deficits. One describes an error that was recognized intraoperatively, the other a situation that was only recognized postoperatively

**Table 3:** Perioperative systemic complications of men and women after aseptic revision surgery; total number of systemic complications for men was *n* = 11 and for women *n* = 22

Systemic Complications	Men (n)	Women (n)	p, statistical effect (phi)
Urogenital:			
- Urinary tract infection*	0	8	0.025, 0.140
- Renal insufficiency	2	1	0.560, −0.063
Cardiac/Vascular:			
- Thrombosis	0	0	1.0, 0.049
- Embolism	0	1	0.149, −0.112
- Apoplex	0	0	-
- Myocardial infarction	2	0	0.149, −0.112
- Arrhythmia	3	0	0.055, −0.137
Pulmonary:			
- Pneumonia	0	2	0.525, 0.069
- Pulmonary edema	1	4	0.625, 0.053
Neurological:			
- Transitory psychotic syndrome	3	6	1.0, 0.019

Overall, no significant sex differences could be found, only urinary tract infection did show a significant difference with a low statistical effect. Also, for the occurrence of multiple surgical und systemic complications during hospitalisation no significant sex differences could be found (surgical complications: mean: 0.32 ± 1.00 vs. 0.43 ± 1.16, *p* = 0.416, phi = −0.104) (systemic complications: mean: 0.26 ± 0.88 vs. 0.22 ± 0.71, *p* = 0.762, phi = 0.038).

**Fig. 1:**
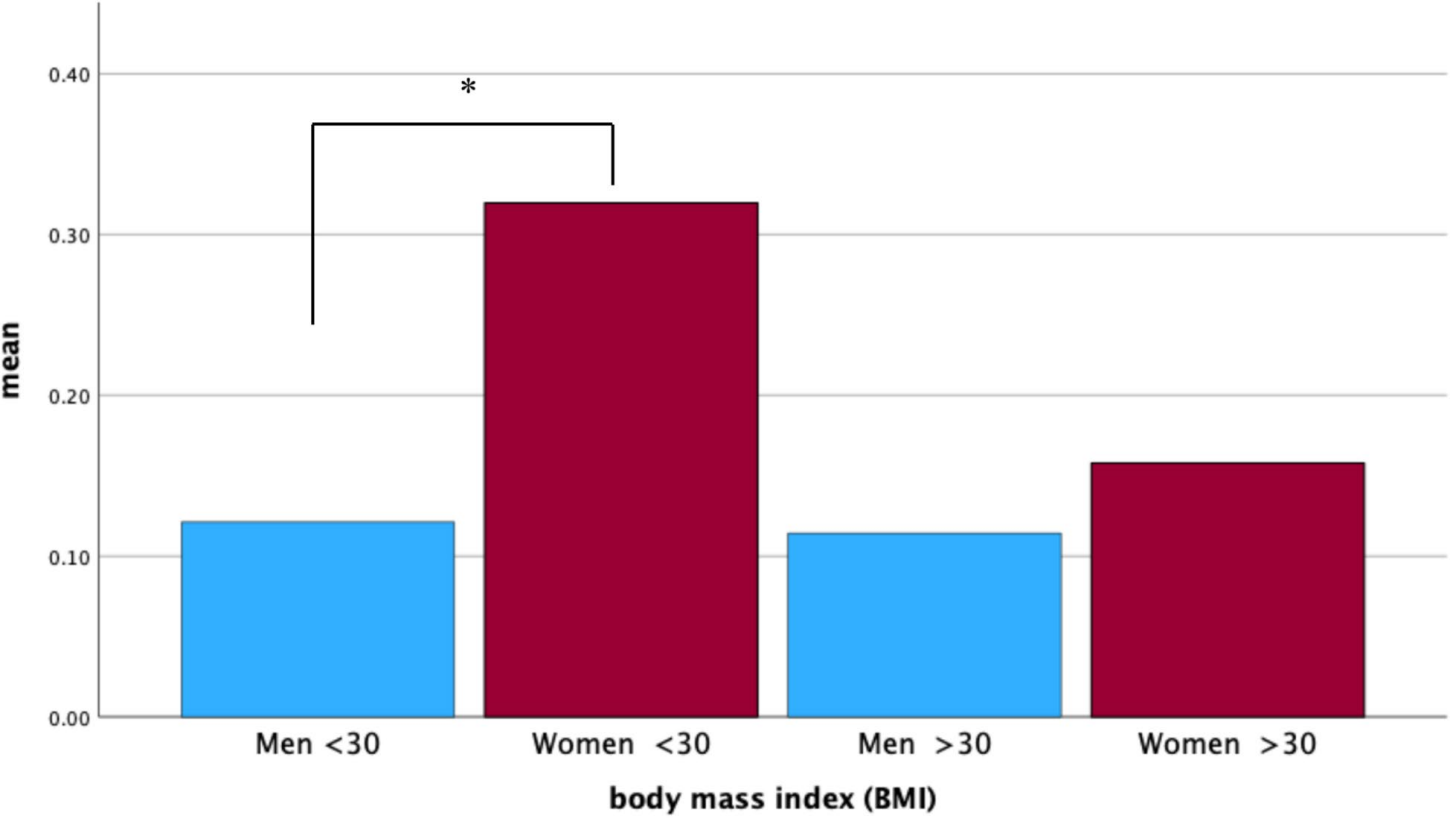
Multiple surgical complications in a patient in BMI + sex groups graphic; * significant difference

The subgroup analyze of sex differences in surgical and systemic complications in combination to the BMI revealed a sex difference for multiple surgical complications in a patient for patients BMI under 30 (Cohen’s d = 0.277; *p* = 0.027, 95%CI 0.37–0.22). No significant differences in the occurrence of multiple surgical complications were found in the BMI group ≥ 30 BMI. (see Fig. 1).

#### Follow-Up

Overall, no significant sex difference in the occurrence of complications in the follow-up was shown (phi = 0.031, *p* = 1.0). In addition, late postoperative infections and persistent nerve damage or coxalgia were not significantly different between sexes (phi = 0.009, *p* = 1.0; phi = 0.018, *p* = 1.0; phi = 0.094, *p* = 0.278). The survival rate after aseptic revision hip arthroplasty was significant different between male and female, as men received Head-Inlay-Changes earlier (*p* = 0.001). In total, there were 20 interventions. More details see Table 4.

**Table 4:** Follow-up interventions

Intervention	Frequency	Percent
No Change	167	64%
Head/Inlay-Change	11	4.2%
Cup + Head/Inlay Change	6	2.3%
Stem + Head/Inlay Change	1	0.4%
Complete Component Change	2	0.8%

Also, three groups of follow-up were built to analyze differences to reduce variances in the results. The groups were divided into three equal numbers of patients, 1) 1–75 days (*n* = 61), 2) 76–150 days (*n* = 53) and 3) ≥ 150 days (*n* = 74). No significance was found for the type of revision between the groups (*p* = 0.600, Z = −0.524). There was a significant difference regarding the occurrence of reinfections (*p* = 0.015, Z = −2.43) with a favor for group 3. For persistent pain, most affected individuals were detected in group 3 (*p* = < 0.001, Z = −3.481).

**Fig. 2:**
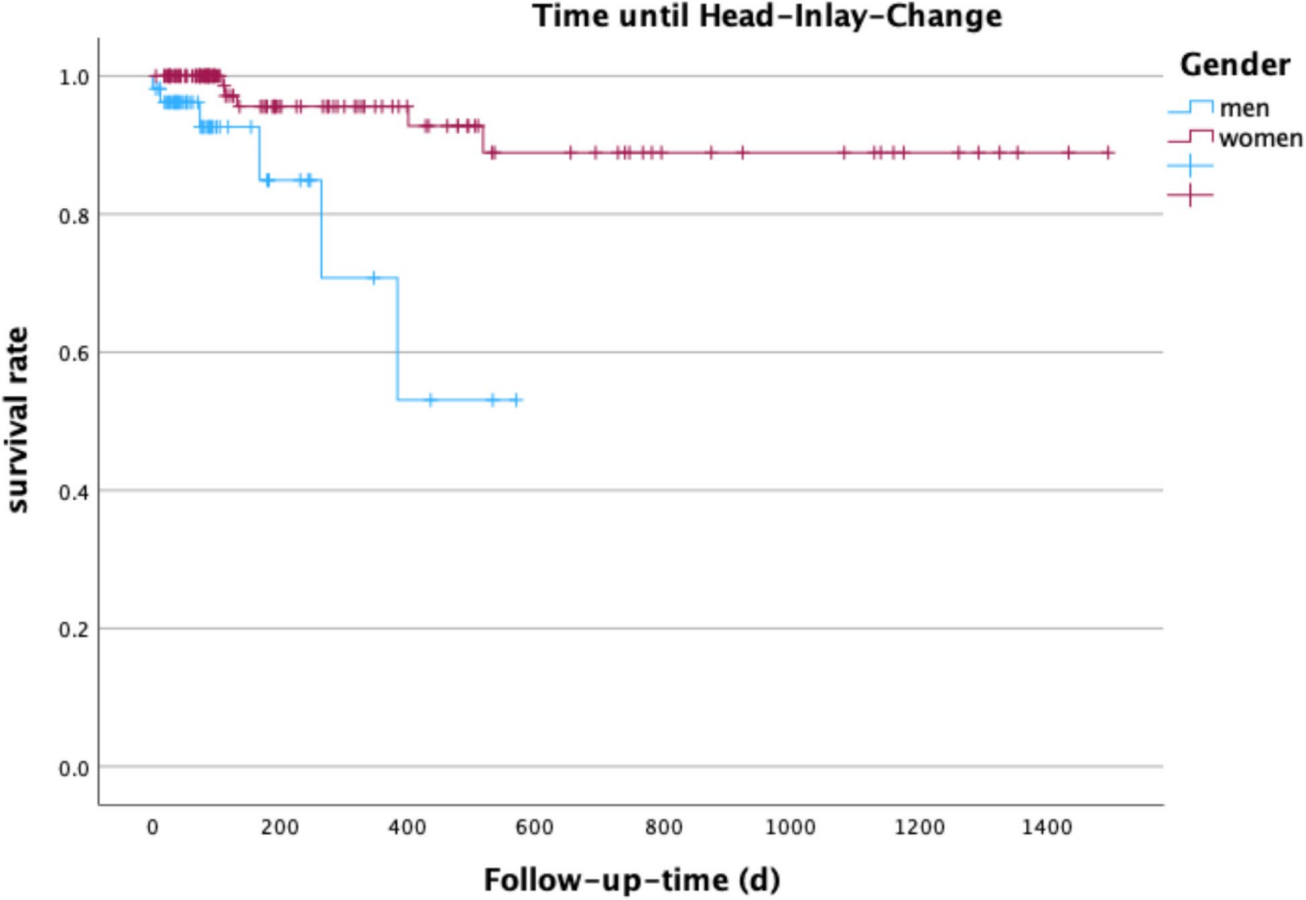
Survival rate of aseptic revision arthroplasty until first surgical intervention

Figure 2 shows the survival rate of men and women until head-Inlay-change.

### Hb Drop

**Fig. 3:**
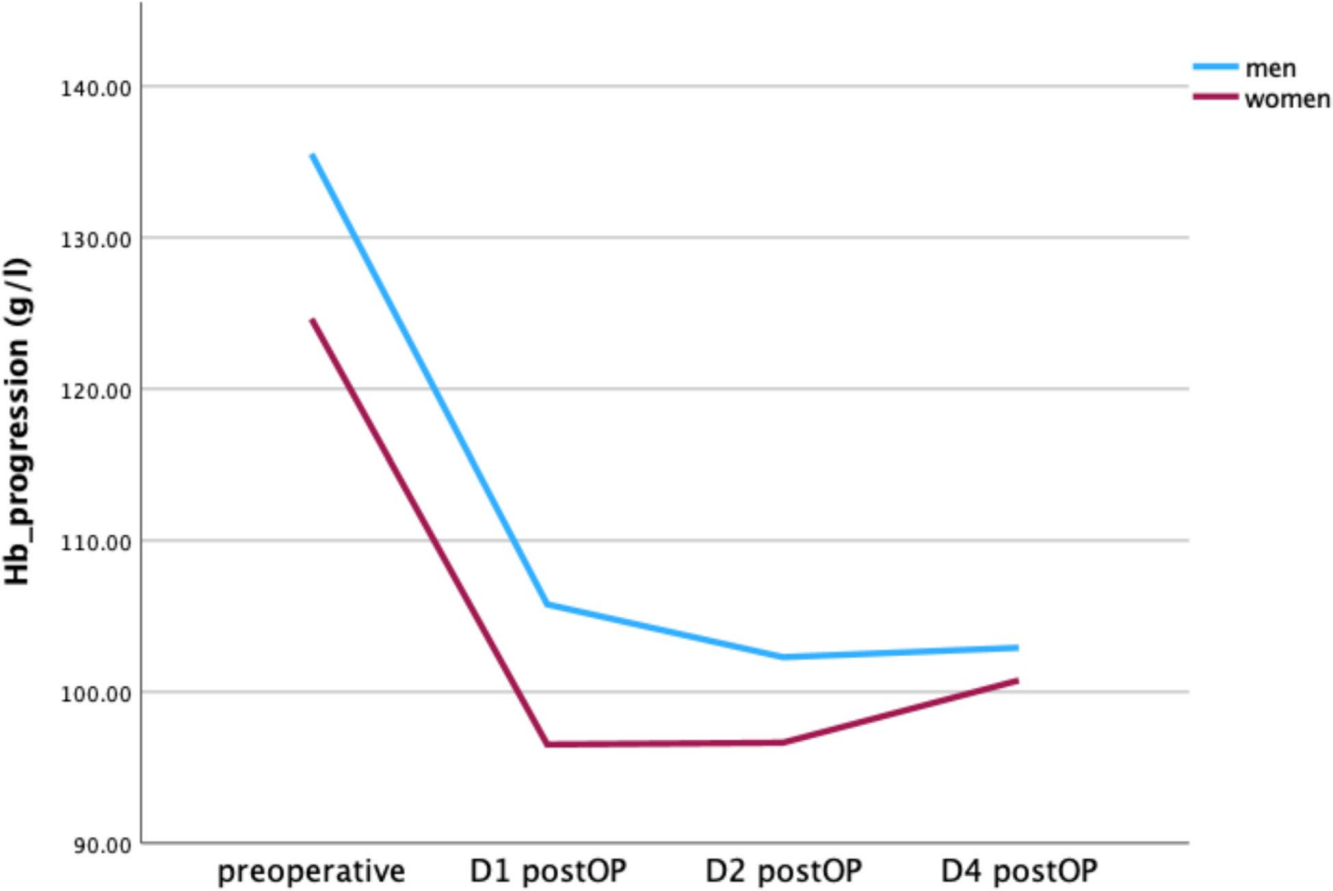
Hb progression between men and women after revision arthroplasty. A significant difference could be revealed for Hb progression (η2 part = 0.092, *p* = <.001). Significant differences were also found between the preoperative, D1 postOP and D2 postOP values (Cohen’s d 0.676, 0.693, 0.472; *p* = 0.001)

Hb was collected preoperatively and on POD (postoperative day) 1, 2, and 4 after surgery. The mean preoperative Hb in men and women was 136.18 ± 16.93 g/L and 124.74 ± 16.91 g/L, respectively. At POD 4, the mean Hb in men and women was 103.02 ± 12.54 and 100.98 ± 13.52 g/L, respectively. Hb progression between men and women did show a significant difference (η2 part = 0.092, *p* = < 0.001) (see Fig. 3).

### Blood units transfused

**Fig. 4:**
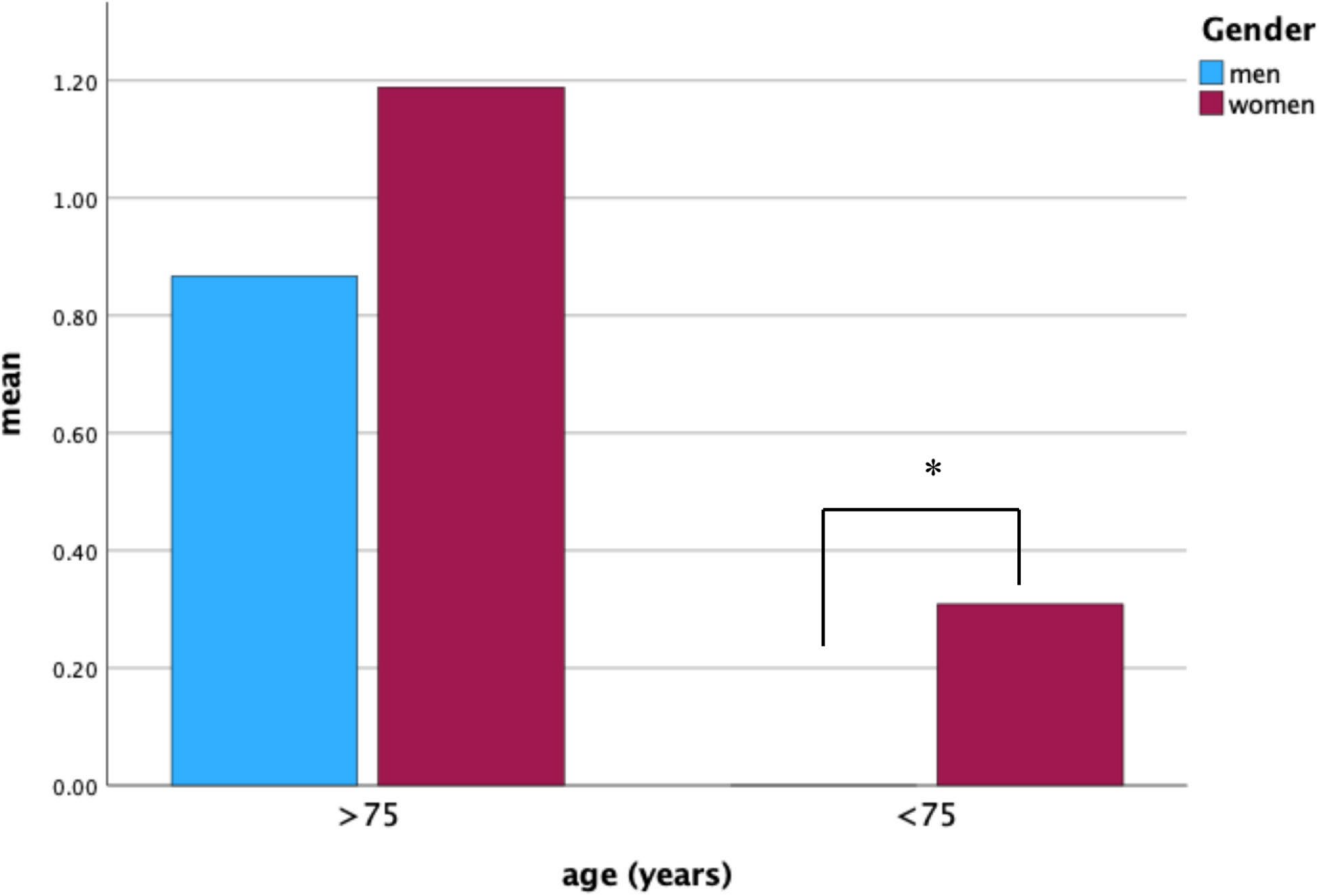
Number of transfusions for sex in the age groups ≤ 75 and ≥ 75 years; *Significant difference

Hb drop under 80 g/l were found in 63 patients, of which 85.7% were women and 14.3% were men (phi = 0.279; *p* = < 0.001). Nevertheless, the rate of postoperative transfused blood units between the sexes was not significantly different (Cohen’s d = 0.196; *p* = 0.144). A comparison of the age groups ≥ 75 years and ≤ 75 years revealed a significant difference in transfused blood units in favor for the old patients (Cohen’s d = 0.642; *p* = 0.001). No significant difference between the sex groups were found for the ≥ 75 years old patients (Cohen’s d = 0.176; *p* = 0.361), but a significant sex difference was found for the ≤ 75 years old patients (Cohen’s d = 0.511; *p* = 0.007). The preoperative Hb and the Hb before discharge were similar for the full and partial component change groups. Only the 1 st and 2nd postoperative days showed a significant difference between the groups, to the disadvantage of the full component change group (1 *p* = 0.014, Cohen’s d = 0.324/2 *p* = 0.027, Cohen’s d = 0.298). Full component changes were more related to blood transfusions than partial component changes (*p* = 0.025, Cohen’s d = 0.349). For details, see Fig. 4.

## Discussion

This study primarily investigated sex differences in systemic and surgical complications, hemoglobin progression, and the rate of blood unit transfusions during hospitalization following aseptic revision THA. Also, follow-up complications like infections or persistent pain were measured. Overall, no clinically relevant perioperative and follow-up sex differences were identified.

Subgroup analyses stratied by BMI and age revealed only minor sex dierences in demographic parameters, including BMI and hospital length of stay in age subgroups, as well as multiple surgical complication occurrence and transfusion rates in age subgroups.

### Comparing theses results with the literature reveals notable differences

For example, female patients were associated with lower odds of mortality with spinal cord tumors or infective endocarditis [27, 28]. Audretsch et al. revealed that females were more related to pelvic fractures than males and women showed a higher affinity for unstable pelvic fractures [29]. Different bone quality of sexes could be a reason sex differences after lower limb arthroplasty, women demonstrated higher risks of wound infections, readmissions, and reoperations in previous studies [30–32]. In this study, for the length of stay, women remained significantly longer in the hospital (Cohen’s d = 0.164, *p* = 0.029), but no significant differences could be found for multiple diseases, or operation time. Interestingly, men ≥ 75 years showed a higher BMI (*p* = 0.003). No significant sex difference in the range of motion was found in this study, but in terms of mobility, men showed a better perioperative outcome (middle rank 119.32 to 136.20, Z = −1.999, *p* = 0.046). This is in line with Peng et al. and Patel et al., who demonstrated prolonged length of stay and more frequent surgery-related complications in women [3, 30]. Possible explanations include difference in pain sensation or muscle strength between the sexes, leading to prolonged rehabilitation. In this study, postoperative pain analysis revealed no significant differences, likely because patients received standardized in-house pain management protocols including baseline analgesics, morphine derivatives, and on-demand medications. Patients requiring reappointment after more than 150 days were more likely to have persistent hip pain at follow-up, suggesting that patients may initially attempt conservative outpatient management before presenting to the clinic.

### No sex differences were found in surgical-related or systemic complications

Only urinary tract infections were more related to women than men. Lamb et al. and others likewise reviewed women as a risk factor for postoperative proximal femoral fractures following total hip arthroplasty [33–35]. Kienzle et al. also showed a significantly increased risk for aseptic loosening after PJI-dependent revision. The authors suggest that theories about different bone metabolism could be the cause [36].

In contrast, Cherian et al. performed a systematic review and found male sex to be more strongly associated with aseptic loosening. The reviewed study included 725 hips, but an overall loosening rate was not reported, as well as the sex distribution of the study group [37]. Notably, in this study, vascular injuries, delayed wound healing, and postoperative neurological deficits were slightly more frequent among women without reaching statistical significance. Given the similar complication proles between males and females, anatomical and physiological factors appear to have limited inuence. Although men received reoperation earlier than women as shown in the Fig. 3, the perioperative outcome was better than in women. A reason could be a different post stationary rehabilitation or a possibly different handling of the revision arthroplasty with different routines or responsibilities in the daily life. Towle et al. 2016 did show the same result after primary hip arthroplasty [38]. A sex specific long-term study could clarify this assumption.

Due to late mobilization and more surgical complications, urinary tract catheters were left longer. That could explain the more frequent occurrence of urinary tract infections after THA in women. This is in line with Robinson et al. and others, who revealed an increased risk for urinary tract infections [39–42].

The existing literature should be expanded to provide a more comprehensive understanding of sex differences in aseptic revision hip arthroplasty outcomes.

Although hemoglobin progression demonstrated sex differences, when hospitalization ended the values assimilated. The literature reports both similar and different postoperative hemoglobin levels after lower limb arthroplasty, depending on the population studied [43, 44]. In this study, no effect on the number of transfused blood units could be shown. No sex differences were found for blood transfusion in general, despite the sexes in the age of ≤ 75 years, were almost only women retrieved blood transfusions. Anyway, the implementation of restrictive blood transfusion protocols, combined with more rigorous assessment of patients' clinical status, has resulted in a reduction of blood transfusion rates over the past several years [45, 46].

Another consideration regarding blood transfusions is the use of drains for aseptic revision arthroplasty. In this hospital every patient gets a drain after revision THA to prevent postoperative hematoma and painful effusion formation. Umer et al. described more blood transfusions and a higher Hb drop for the drained patients [47]. On the other hand, no significant differences in total blood loss and blood transfusion between the no-drainage and drainage groups were shown [48]. The use of drainages in orthopedic surgery is remains controversial. The experts’ opinions differ significantly.

This study is limited principally by its retrospective design, with the follow-up period being unscheduled and dependent on individual patient re-appointments, potentially leading to incomplete follow-up data. In-hospital data quality occasionally suffered due to loss of archived information. No fixed age limit was established for inclusion, and only sex-based analyses of perioperative complications were conducted, which may introduce bias despite matching for age, BMI, comorbidities, and ASA score. Furthermore, the study cohort focused exclusively on patients with uncemented implants to maintain group homogeneity, but this excludes a relevant proportion of revision arthroplasty cases with cemented implants and limits generalizability.

## Conclusions

This study identified no significant statistically or clinical sex-related differences in perioperative complications and blood transfusion patterns after aseptic revision total hip arthroplasty. Although minor sex differences in demographic factors were defined, theses significant results should be viewed with caution or reserve when drawing conclusions regarding clinical relevance. The number of perioperative and follow-up complications is low and the analyze group is specified on only primary uncemented arthroplasties. Future prospective studies with scheduled follow-up and inclusion of cemented implant patients should address these gaps, allowing more representative and robust assessment of sex-specific complications and transfusion requirements after aseptic revision THA.

## Data Availability

The datasets used and analyzed during the current study are available from the corresponding author on reasonable request.

## Abbreviations

ASA: American Society of Anesthesiologists
BMI: Body Mass Index
cCRA: Complete Component Revision Arthroplasty
CI: Confidence Interval
d: Cohen’s d (effect size)
EK: Erythrocyte Concentrate (RBC unit)
Hb: Hemoglobin
LOS: Length of Stay
n: Number
p: P-value (statistical significance)
pCRA: Partial Component Revision Arthroplasty
POD: Postoperative Day
ROM: Range of Motion
SD: Standard Deviation
THA: Total Hip Arthroplasty
UTI: Urinary Tract Infection
VAS: Visual Analogue Scale
